# Interpersonal problem behavior and low back pain

**DOI:** 10.1371/journal.pone.0207173

**Published:** 2018-11-15

**Authors:** Constanze Borys, Steffi Nodop, Christoph Anders, Robin Tutzschke, Hans Christoph Scholle, Andrea Thomas, Uwe Altmann, Bernhard Strauss

**Affiliations:** 1 Institute of Psychosocial Medicine and Psychotherapy, University Hospital Jena, Friedrich-Schiller-University Jena, Jena, Germany; 2 Division Motor Research, Pathophysiology and Biomechanics, Clinic of Trauma, Hand and Reconstructive Surgery, University Hospital Jena, Friedrich-Schiller-University Jena, Jena, Germany; Teesside University, UNITED KINGDOM

## Abstract

**Objective:**

The theory of interpersonal problem behaviour (IPB) provides a more fundamental framework for understanding the psychosocial aspects of pain. The present study focused on the IPB, based on the Interpersonal Problem Circumplex (IPC), in persons with low back pain and its association with pain, psychological characteristics, and health care utilisation.

**Methods:**

In a cross-sectional design, individuals with back pain (N = 88) and healthy control persons who matched by age, gender, and educational level (N = 88) were compared with regard to IPB. Furthermore, back pain patients classified by their IPB (N = 24 low, N = 48 moderate, N = 16 high) were compared regarding pain, depression, catastrophising, and health care utilisation.

**Results:**

In comparison to the healthy reference sample, a significant difference in the interpersonal problems of the low back pain group, with a tendency towards being overly ‘introverted’, ‘exploitable’, and ‘subassertive’, was revealed. In the back pain group, participants with elevated IPB showed significantly higher levels of pain intensity, functional disability, depression, catastrophising, and health care utilisation than participants with IPB in the normal range.

**Conclusion:**

Application of the Interpersonal Circumplex Model can help to characterize a subgroup of persons with low back pain. Increased general interpersonal problems are associated with elevated burden in pain-related, psychological, and health care-related variables. Future research should focus on the treatment opportunities for this subgroup, as well as on the influence of interpersonal problems during the course of back pain.

## Introduction

Low back pain is a common health problem in developed countries, with estimated prevalence rates between 2.0% and 46.5%, depending on the definition of pain, sampling procedures, or national differences [1]. Psychological and social factors were revealed to be important predictors, especially for the transition from acute to chronic pain conditions [2]. The expression of pain may be aimed at encouraging others to help and could therefore be regarded as a social-communicative event. Individuals suffering from low back pain can influence their social environment via reduced participation and/or limited communication (e.g., loss of parts of their functional ability, impaired mood, or social retreat). However, reactions of family members, colleagues, and even society can also moderate pain behaviour and the experienced pain intensity, as well as the persistence of low back pain [3]. Their reactions can vary between offering and refusing to help [4, 5]. The influence of spousal interaction on the pain condition has been especially well studied in recent decades. Results have showed weak or no associations of marital satisfaction and pain, but spousal support and responses in the context of chronic pain were related to pain severity, physical disability, and depression [6].

A study by Turk and Rudy [7] aimed to integrate affective, cognitive, and behavioural aspects of pain to allow classification of patients. They identified an empirically derived taxonomy with three groups—‘dysfunctional’, ‘minimizers/adaptive copers’, and ‘interpersonally distressed’—on the basis of the Multidimensional Pain Inventory (WHYMPI) [8]. Patients in the interpersonally distressed group reported a lack of support from their families and significant others. Furthermore, this group was characterised by elevated pain-related distress in combination with elevated psychological distress and increased health care utilisation [7]. This three-group taxonomy was replicated several times [9].

The complex interplay between social interaction and pain is not fully understood yet. The roles of certain variables influencing social interaction, like interpersonal problem pattern, should be addressed.

### Interpersonal problem circumplex model (IPC)

The theory of interpersonal problem behaviour (IPB), which originated in personality and psychotherapy research, provides a fundamental framework for further understanding the role of the interpersonal context in pain. The interpersonal problem circumplex (IPC) model [10–12] is a comprehensive theoretical model for conceptualising and measuring interpersonal behaviour, attitudes, and processes. The basic idea behind it is to describe a system of relationship patterns in order to better understand adaptive and maladaptive interactions. As described above, maladaptive interactions in the marital context, for example, are associated with elevated pain and psychosocial symptoms [6]. Furthermore, understanding interaction processes can facilitate patient–provider communication and may therefore improve pain management, such as with regard to shared medical decision-making [13].

**Fig 1:**
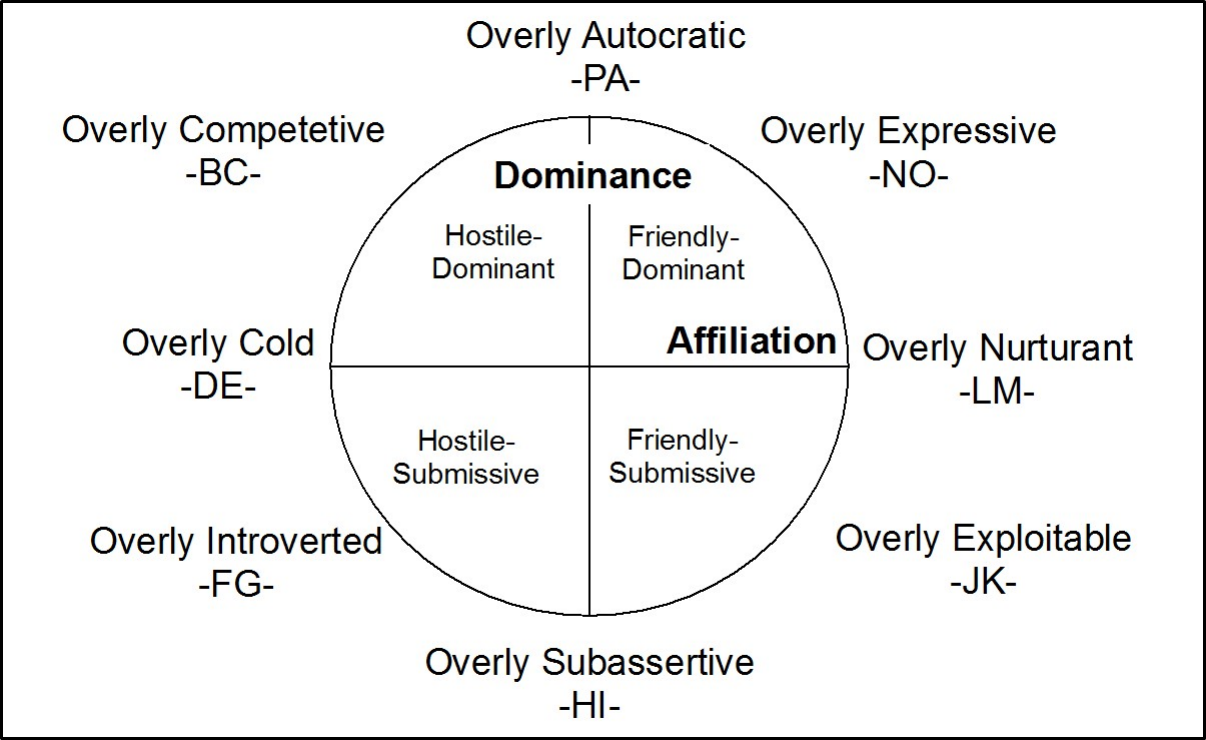
Interpersonal problem circumplex (IPC), based on Horowitz et al. [15], modified version.

The IPC model is based on various assumptions. One of them is the idea that any interpersonal behaviour in humans can be described along the axes of a two-dimensional space, with affiliation (communion) as the first dimension and dominance (agency) forming the second dimension (Fig 1). Alden et al. [14] described a circumplex version of the IPB model based on the Inventory of Interpersonal Problems (IIP) [14–16]. This version is comprised of eight problem categories (octants) reflecting a specific combination of dominance and affiliation. All IPBs can be arranged on the circumplex model in a specific way (cf. [17]. The IPC model has been empirically confirmed by a range of different authors [11, 12, 18, 19].

### Interpersonal problem circumplex model in pain research

Based on the Interpersonal Circumplex Model, it is possible to screen subjects for elevated IPB (total score), and it allows identification of main problem categories (octants) in the interpersonal behaviour. Thus far, research based on the Interpersonal Circumplex Model in the field of low back pain is rare. A Norwegian study revealed that individuals suffering from pain disorders (DSM-IV, axis I: 307.80) typically described themselves as ‘overly introverted’, ‘overly exploitable’, and ‘overly nurturant’ [20]. A German study compared persons who suffered from different chronic pain conditions and attended a university pain clinic to the normative data of the IIP [21]. This study revealed significant differences in almost all octants. Persons suffering from chronic pain were more likely to be classified as ‘overly subassertive’, ‘overly exploitable’, and ‘overly nurturant’ [21].

### Aim of the study

The aim of the present study was to investigate the IPB of individuals with low back pain. First, the IPB of back pain patients was compared to healthy controls. Second, back pain patients were classified in three groups depending on their IPB (low: below the average, moderate: similar to the average, high: above the average) and compared with each other regarding pain, and previous health care utilisation. Psychological characteristics were compared focused on catastrophizing and depression as one of the most prevalent comorbidities in patients with low back pain to allow comparisons with the results of similar studies.

Hypotheses:

1. Interpersonal pattern: When compared to a healthy reference group, individuals with back pain are more likely to be classified as ‘overly introverted’ (FG), ‘overly subassertive’ (HI), ‘overly exploitable’ (JK), and ‘overly nurturant’ (LM).
2. Interpersonal distress: In the back pain group, participants with elevated IPB (high IIP stanine values 7–9) will show higher levels of pain intensity, functional disability, psychological distress (depression, catastrophising), and health care utilisation as compared to participants with typical interpersonal behaviour (moderate IIP stanine values 4–6).

## Methods

### Participants

All participants signed a written informed consent form prior to the investigation. The study was approved by the local ethics committee of the Friedrich-Schiller-University (2687-11/09) and therefore fulfils the ethical standards required for investigations of humans, according to the Declaration of Helsinki.

Back pain group: There were 88 participants with low back pain who took part in this study. Participants were recruited for a back-school intervention study via a newspaper advertisement and were consecutively included if they met the following inclusion criteria:

- chronic non-specific low back pain (duration > three months; no restriction on the reported minimum level of pain),
- aged between 18 and 69 years,
- sufficient understanding of the German language.

Exclusion criteria were:

- radicular symptoms,
- active rheumatoid disease,
- senile osteoporosis,
- spinal surgery,
- long-term use of pain-reducing medication.

After the final measurement (12-month follow-up), participants received a compensation of 10 €.

The mean age of the 88 participants was 47.5 (SE = 1.38, range: 19–70) years. The level of education was relatively high, and 68.2% of participants received more than 10 years of school education. The proportion of women predominated at 68.2% (N = 60). At the time of the survey, 79.5% (N = 70) of the participants were employed. The remaining participants were either students or retired.

During the first examination, the average pain duration was 95.0 (SE = 13.3) months, with an average pain intensity level of NRS (0–10) = 3.1 (SE = 0.2 and a range from NRS = 0 to NRS = 9). Most participants (N = 49, 55.7%) reported persistent pain. The physical functional disability in the areas of work, family, and everyday activity was NRS (0–100) = 17.5 (SE = 2.0).

Healthy reference group: The healthy reference group was drawn from the normative sample of the IIP-32. The collection of this normative sample was carried out by the Opinion Research Institute ‘USUMA’ (Berlin) within face-to-face interviews in the year 2007. In the final validation, a representative population sample of N = 2515 was included [17]. For our study, we matched 88 healthy controls from this normative sample of the IIP-32 to participants of the intervention group by age (±2 years), sex, and educational level (years of school education). In order to ensure a good level of health status in the reference group, we included only individuals who had rated their general health with scores of at least 3 on a scale from 0 (very bad) to 4 (very good). If more than one healthy individuals fulfilled the matching characteristics of one patient, one of these individuals was randomly selected, finally leading to a one-to-one matching of patients and healthy controls.

The mean age of the 88 healthy participants was M = 47.7 (SD = 12.9) years. They showed a high level of education (69.4% with more than 10 years of school education), and the proportion of women predominated at 68.2% (N = 60).

### Procedure

#### German New Back School

In the basic evaluation study, the effects of the German New Back School on pain, functionality, and the performance of activities of daily living in users with chronic back pain were evaluated. The New German Back School integrates components of the classical Back School (exercise) and education based on psychological and social aspects. The back pain group participated in the Back School, with 12 one-and-a-half-hour theoretical and practical trainings held once a week [22].

#### Study design

The evaluation study of the German New Back School was conducted as a longitudinal study with five assessment-time points. Due to the waiting-list design, materials were administered to participants at the beginning of the three-month waiting period prior to the Back School as well as at the beginning and end of the Back School. They were also surveyed three and 12 months after their time at the Back School.

The present analyses are focused on the baseline variables assessed in the evaluation study mentioned above. Additionally, Data for the healthy reference group were drawn from the German normative sample of the IIP-32, which was based on a representative cross-sectional survey (see above). Socio-demographic data and IPB were assessed via standardised questionnaires.

### Measures

German Pain Questionnaire of the German Section of the IASP (DSF) [23]: The DSF was used for assessing socio-demographic characteristics (e.g., age, gender, and educational level), pain-related characteristics (e.g., pain intensity, chronic pain grade, and disability score), and health care utilisation.

Numerical Rating Scale (NRS): Back pain intensity was assessed by NRS 0–10 (0: ‘no pain at all’ to 10: ‘strongest imaginable pain’) in the last three months.

Chronic Pain Grade and Disability Score [24], (German version [25]): The disability score by Von Korff et al. was used to assess functional disability in the last three months [24]. This score is calculated as the average of the three functional areas—work, family, and everyday life (NRS = 0–10)—and multiplied by 10 [24, 25].

Pain duration: The duration of pain was assessed as the number of months since the first appearance of back pain.

Pain persistence: Participants were asked to indicate their back pain as persistent pain or pain attacks with pain-free periods between.

Health care utilisation:

- Number of consulted medical profession groups: Participants were asked to indicate which specialists they consulted as a result of their back pain symptoms from a list of 11 medical professions (e.g., general practitioner, neurologist, orthopaedist, psychiatrist, and pain therapist) [23]. The reference period is since the onset of back pain.
- Number of types of treatments used: Additionally, participants had to indicate the use of different treatment types from a list of 13 different treatments (e.g., massage treatments, medication, and body exercises) [23]. The reference period is since the onset of back pain.

The assessment of the psychological characteristics was carried out using established standardised questionnaires with adequate to good psychometric characteristics.

Hospital Anxiety and Depression Scale (HADS [26], German version [27]): The HADS was developed to determine the levels of anxiety and depression in individuals with physical health problems. In the present study we focused only on depression as one of the most prevalent comorbidities. Only the depression scale was used. The depression scale contains seven items (4-point Likert scale). Sum scores of ≤ 7 are considered normal [27].

Coping Strategies Questionnaire (CSQ [28, 29], German version [29]): The CSQ is a self-report measure for the detection of pain-related coping strategies. Only the catastrophising scale was selected for the present study. Participants were asked to indicate the frequency of the strategy used during back pain episodes on a 7-point Likert scale [29].

Inventory of Interpersonal Problems (IIP-64 [15, 16], German version IIP-32 [17]): The IIP was constructed in a psychotherapy setting and allows persons to describe how much they suffer from specific difficulties when dealing with other people. Examples of items describing problem behaviour are ‘I try to control other people too much’ and ‘I tell personal things to other people too much.’ Other items begin with ‘It is hard for me…’ (e.g., ‘It is hard for me to show affection to people’).

A recent 32-item short version of the IIP was used for this study (IIP-32 [17]). The IIP-32 is a self-report measure that assesses eight domains of interpersonal problems. Problematic interpersonal experiences are summarised in eight subscales (octants), which can be plotted on the IPC along the dimensions of ‘dominance’ and ‘affiliation’ [14]. The scale designations are based on the alphabet and arranged counterclockwise around a circle, where PA is the beginning as well as the end of the circle (Fig 1). Sum scores range from 0 to 16 for each octant (total sum score 0–128). Results in the present study are based upon the average score for each octant.

### Statistical procedures

Missing data were multiple imputed (10 estimations) using SPSS (Version 19.0). Statistical analyses reported in this article are based on the pooled results of the estimated 10 data files. Due to the directional hypotheses (see above), all statistical tests were one-tailed. The level of significance was p ≤ .05. To avoid spurious positives in multiple comparisons, a Bonferroni–Holm procedure was applied [30].

At first, a series of t-tests was applied for the comparison of Back School participants and healthy controls and comparisons of different interpersonal problems in the Back School group. For the comparison of Back School participants with elevated vs. normal IPB, we recoded the IIP total score into stanine values, depending on age and gender (stanine value range: 1–9). The age and gender cut-off values used for recoding were based on a large representative sample by Horowitz et al. [31]. In that way, a potential age and gender bias was eliminated. Stanine values of 4–6 were considered to be normal interpersonal behaviour, whereas values of 7–9 were defined as elevated levels of interpersonal behaviour. Pain, depression, catastrophising, and medical treatment utilisation measures were compared between both groups (stanine 4–6 vs. stanine 7–9) using t-tests.

Stanine values of 1–3 were also outside the normal range. A specific interpretation is actually not clarified. Individuals with these scores were therefore considered separately [31]. Nevertheless, to minimise the loss of information, mean values, standard error, and comparison to the normal group (stanine 4–6) were reported in the results section. The Statistical Software Package for Social Sciences (SPSS version 19.0) was used for the data analysis.

## Results

### Hypothesis (1): Interpersonal pattern of Back School participants

**Fig 2:**
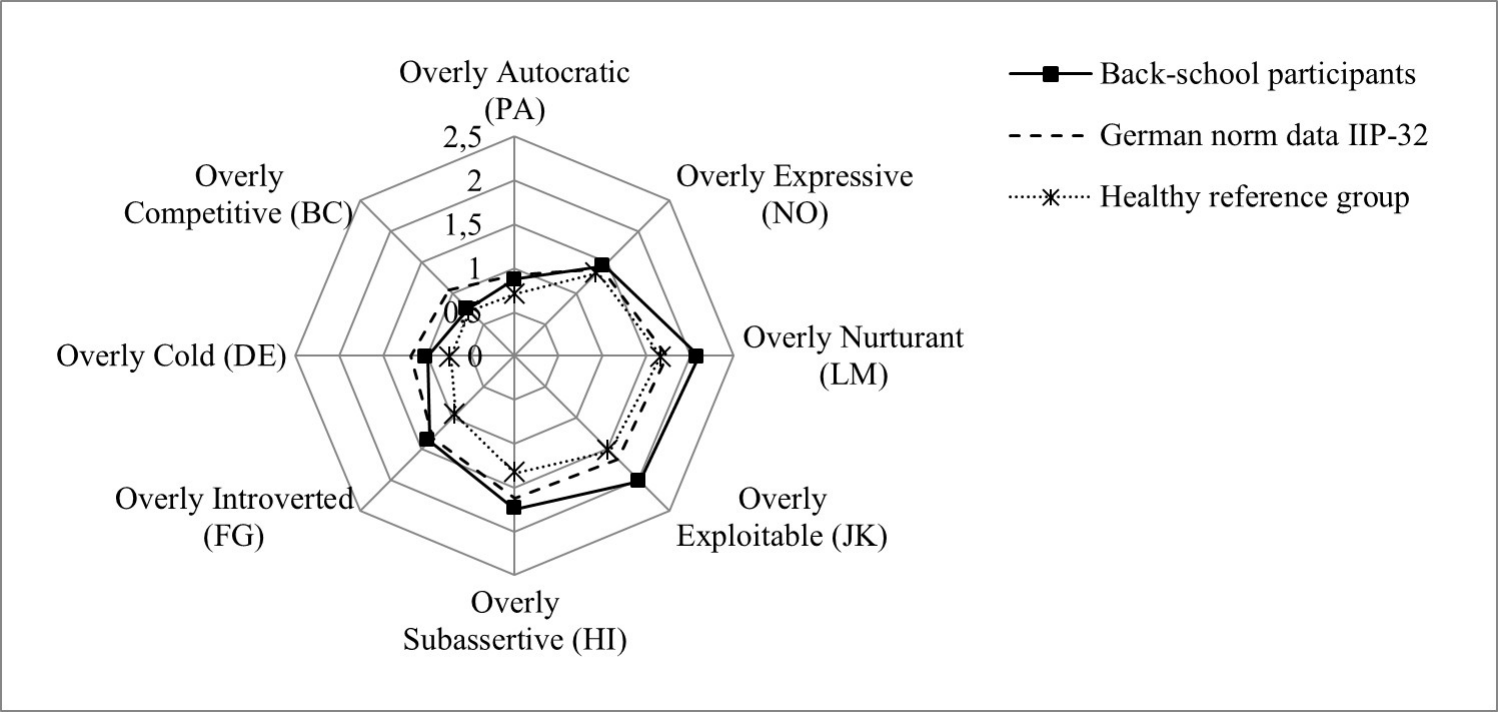
IIP profile (means) of participants of the New German Back School (N = 88), matched reference group (N = 88), and German norm data.

The comparison with the matched healthy sample (reference group) showed significantly higher scores for the octants ‘overly introverted’ (FG), ‘overly subassertive’ (HI), and ‘overly exploitable’ (JK). The effect sizes of average differences are small (introverted and subassertive) to respectively moderate (exploitable). Differences in all other octants failed to reach significance (Fig 2, Table 1).

**Table 1:** Mean (M) and standard error (SE) of the IIP-32 octant values and group differences.

Octant IIP-32	Back School group (BS) N = 88 M (SE)	Healthy reference group (HR) N = 88 M (SE)	M_Diff_ BS-HR	p-value (one-sided tests)	Cohen’s d
**Overly Autocratic (PA)**	0.89 (0.15)	0.70 (0.07)	-0.19	0.017	.17
**Overly Competitive (BC)**	0.72 (0.11)	0.72 (0.07)	0.00	0.050	.00
**Overly Cold (DE)**	0.94 (0.11)	0.73 (0.07)	-0.21	0.013	.24
**Overly Introverted (FG)**	1.31 (0.10)	0.95 (0.07)	-0.36	0.006*	.45
**Overly Subassertive (HI)**	1.67 (0.09)	1.34 (0.08)	-0.33	0.007*	.42
**Overly Exploitable (JK)**	1.93 (0.08)	1.53 (0.08)	-0.40	0.006*	.54
**Overly Nurturant (LM)**	1.91 (0.08)	1.67 (0.09)	-0.24	0.010	.30
**Overly Expressive (NO)**	1.30 (0.09)	1.34 (0.08)	0.04	0.025	.05
**IIP-32 Total Score**	**1.33 (0.06)**	**1.12 (0.05)**	**0.20**	0.008*	**.41**

Note: Means compared with the use of t-tests for independent samples.

*significant results according to Bonferroni–Holm procedure (one-tailed t-tests)

### Hypothesis (2): Interpersonal distress (comparison of stanine groups)

The categorisation in stanine value-based groups revealed that half of the participants (n = 48, 54.5%) reported IPBs within the standard range (stanine value: 4 (n = 18), 5 (n = 16), and 6 (n = 14)). A total of 16 participants (18.2%) were classified above the normal range (stanine value: 7 (n = 9), 8 (n = 5), and 9 (n = 2)), and 24 participants (27.3%) were classified below the normal range (stanine value 1 (n = 5), 2 (n = 7), and 3 (n = 12)).

The means for pain, psychological variables, and health care utilisation, depending on the stanine group (IIP-32 total score), are summarised in Table 2. Furthermore, the comparison between stanine group (4–6) and stanine group (7–9) is listed in Table 2.

**Table 2:** Averages of pain, psychological distress, and health care utilisation depending on stanine value-based groups and group comparisons.

			Stanine values IIP-32_total score_		M_Diff_ (4–6) vs. (7–9)	p-value	Cohen’s d	M_Diff_ (4–6) vs. (1–3)	p-value	Cohen’s d
		(1–3) n = 24 M(SE)	(4–6) n = 48 M(SE)	(7–9) n = 16 M(SE)						
PAIN	**Pain intensity (NRS)**	3.2 (0.4)	2.8 (0.2)	4.0 (0.6)	-1.20	0.014*	.72	-0.4	0.151	.71
	**Functional disability (disability score,** **Von Korff)**	19.0 (4.3)	13.8 (2.2)	28.1 (5.4)	-14.30	0.002*	.90	-5.2	0.115	.84
	**Pain duration (months)**	134 (32.5)	77 (16.4)	91 (24.2)	-14.70	0.307	.13	-57	0.044	.13
PSYCHO-LOGICAL	**Catastrophising (CSQ)**	4.6 (1.4)	4.3 (0.7)	8.3 (1.4)	-3.90	0.003*	.80	-0.3	0.415	.80
	**Depression (HADS)**	2.4 (0.6)	3.5 (0.6)	6.4 (0.8)	-2.90	0.001*	.74	1.1	0.073	.73
HEALTH CARE USE	**Consulted medical profession groups** **(range 0–11)**	1.5 (0.2)	1.3 (0.1)	1.8 (0.3)	-0.56	0.025	.60	-0.2	0.264	.59
	**Different types of treatment used** **(range 0–13)**	3.5 (0.5)	2.1(0.3)	3.6 (0.5)	-1.44	0.004*	.73	-1.4	0.005*	.72

Note: M = average, SE = standard error, p = p-value of one sided t-test, M_diff_ = difference of two group means, Cohen’s d = effect size. Means compared with the use of t-tests for independent samples.

*significant results according to Bonferroni–Holm procedure

With the exception of pain duration, persons with elevated interpersonal problems (stanine values 7–9) were severely more impaired than the group with normal levels: pain intensity (M_Diff_ = -1.2, d = .72), physical disability (M_Diff_ = -14.3, d = .90), catastrophising (M_Diff_ = -3.9, d = .80), and depression (M_Diff_ = -2.9, d = .74) reached statistical significance. Increased interpersonal problems were also connected with a significantly higher sum of types of treatment used (M_Diff_ = -1.4, d = .73). The number of consulted medical profession groups failed to reach significance (M_Diff_ = -0.6, d = .60). The effect sizes were large for physical disability and catastrophising and moderate for all other variables.

The comparison between stanine group (4–6) and stanine group (1–3) is listed in Table 2. With the exception of the sum of types of treatment used (M_Diff_ = -1.4, d = .73), persons with stanine values between 1 and 3 showed no significant differences compared to the group with normal levels: pain intensity (M_Diff_ = -0.4, d = .71), physical disability (M_Diff_ = -5.2, d = .84), pain duration (M_Diff_ = -57, d = .13), catastrophising (M_Diff_ = -0.3, d = .80), and depression (M_Diff_ = 1.1, d = .73). The number of consulted medical profession groups also failed to reach significance (M_Diff_ = -0.2, d = .59).

## Discussion

The presented study examined the relationship among low back pain, IPB, and health care utilisation. Our first main finding was that participants with low back pain tend to be overly ‘introverted’, ‘exploitable’, and ‘subassertive’ compared with healthy controls. Furthermore, we classified low back pain participants into three subgroups depending on their interpersonal problem scores into low (stanine value 1–3), normal range (stanine value 4–6), and high (stanine value 7–9), whereby the normal range group served as the reference group. Our second main finding was that low back pain participants with high interpersonal problem scores (stanine values 7–9, N = 16) showed higher levels of pain intensity, functional disability, depression, catastrophising, and health care utilisation than low back pain participants in the normal range of interpersonal problems (stanine values 4–6).

The first finding underlines that low back pain is not only associated with psychological aspects (e.g., higher depression), but also with psychosocial aspects (e.g., maladaptive interaction pattern). It can be assumed that patient–physician communication is affected by the patient’s IPB, which can result in sub-optimal diagnosis (e.g., due to overly subassertive interpersonal behaviour, health problems were not communicated), sub-optimal fit of treatment and patient’s needs (e.g., due to overly subassertive interpersonal behaviour, the patient´s concerns and wishes were not communicated), and dissatisfaction of the patient with the physician [32]. Furthermore, the IPB of low back pain patients underlines the need for multi-modal therapies in which psychological and somatic therapies take place.

Our first main finding corresponds to a few international studies that have investigated the interpersonal problem pattern in persons with chronic pain, based on the Interpersonal Circumplex Model. These studies almost uniformly indicate a profile of interpersonal problems with peaks in the ‘overly introverted’, ‘overly subassertive’, ‘overly exploitable’, and ‘overly nurturant’ octants [20, 21, 33, 34]. Our study showed comparable interpersonal patterns with significantly elevated scores for the octants ‘overly introverted’, ‘overly subassertive’, and ‘overly exploitable’. Persons with the mentioned interpersonal behaviour represent difficulties in saying ‘no’ to others and confronting others, and they tend to be exploited by them (‘overly exploitable’). They also show problems of being aggressive towards others or confronting them with problems, and they see themselves as slightly self-conscious in the presence of others (‘overly subassertive’). ‘Overly introverted’ behaviours relate to problems in socialising with other people and describe difficulties in showing their own feelings [31]. Furthermore, low back pain patients have problems accepting and trusting others and being socially avoidant, expecting the physician at the beginning of pain therapy to be less competent [32]. The possibility that those interpersonally distressed patients rated their course of low back pain at the one-year follow-up negatively was even higher [32]. Treatments of low back pain patients should take this into account. Patients with elevated IPB, for example, might benefit more from combined pain-related therapy and interpersonal-based psychotherapy. Especially overly socially avoidant interpersonal behaviour can be treated successfully by interpersonal-based group therapy [35]. Wittingham [22] for example, developed a brief, semi-structured process group approach targeting reduction in interpersonal distress (Focused Brief Group Therapy, FBGT). Focus lies on specific interpersonal behaviours that will decrease the patient’s level of interpersonal distress. Patients with socially avoidant behaviour in particular could improve due to this group therapy. Concurrent individual therapy is less likely to target this kind of change specifically [36].

Our second finding underlines the importance of characterising subgroups in patients with low back pain. From a clinical point of view, it is of interest to what extent individuals need appropriate flexibility of their interpersonal behaviours and how this might interfere with pain symptoms. Participants in the group with lower IPB (stanine values 1–3) reported the highest pain duration and the lowest levels of depression. Depressive symptoms are especially known to be related to IPB, as well as to higher pain and disability levels. The levels of pain intensity, bodily function, catastrophising, and health care utilisation were in between the normal group and the group with elevated IPB. It can be hypothesised that persons in this group are more able to accept pain and disability and cope well. It is also possible that persons in this group act with more pain-suppressive behaviour, as hypothesised in the Avoidance-Endurance Model [37].

Participants in the subgroup with elevated interpersonal problems (stanine values 7–9) showed significantly reduced bodily function and higher levels of pain intensity, psychological distress, and health care utilisation (sum of the types of treatment used). These results are consistent with those of Turk and Rudy [7], who supposed that among persons with pain, some might show specific interpersonal distress combined with higher pain intensity levels and reduced emotional functioning. These interpersonally distressed persons were also more likely to receive less social support, and they tended to report a more catastrophising coping style [7]. Further studies are needed to explore causality. It remains unclear if a higher burden of pain causes IPB or vice versa. Nevertheless, one can assume that IPB in patients with low back pain is an additional burden and should be considered in low back pain therapy, as mentioned above.

### Limitations and strengths of the study

The results of the present study do not allow for any statement about causality, such as whether the interpersonal patterns are to be considered as a result of the pain or if they exist independently from pain. Longitudinal surveys are needed to better understand the temporal and causal relations between interpersonal behaviour problems and pain. Furthermore, the generalisability of the results is potentially limited, since the relatively small opportunity sample consisted of persons with a comparatively low burden of low back pain who participated voluntarily in the study. The health status of our derived reference group (IIP-32 normative sample) was categorised on the basis of the self-assessment of respondents. Selecting individuals with a self-reported good health status can substantially, but not completely, exclude the presence of pain in the reference group.

Despite several limitations, our study revealed important new information on predominant interpersonal problem pattern of persons with low back pain by using an instrument based on the interpersonal theory of personality [38]. This might lead to more differentiated management (especially with respect to the concepts of complementarity and reciprocity) for these individuals in both prevention and therapy. Furthermore, a subgroup with elevated IPB among the participants with low back pain was revealed. As reported earlier by Turk and Rudy [7], based on the WHYMPI, this interpersonally distressed group showed higher pain-related and psychological distress.

## Supporting information

S1 Dataset (SAV)
